# Synthesis of Modified Silver- or Copper-Loaded Silica Nanocarriers as a Tool for Curcumin Delivery

**DOI:** 10.3390/nano16181163

**Published:** 2026-09-15

**Authors:** Anna Sierosławska, Anna Borówka, Izabela Korona-Głowniak, Anna Rymuszka, Jakub Ciepielski, Andrzej Górski

**Affiliations:** 1 Faculty of Medicine, The John Paul II Catholic University of Lublin, 20-708 Lublin, Poland; anna.rymuszka@kul.pl (A.R.); jakub.ciepielski@kul.pl (J.C.); andrzej.gorski@kul.pl (A.G.); 2 Faculty of Pharmacy, Medical University of Lublin, 20-093 Lublin, Poland; izabela.korona-glowniak@umlub.edu.pl

**Keywords:** curcumin, mesoporous silica nanoparticles, cytotoxicity, cancer cells, antimicrobial activity

## Abstract

Mesoporous silica nanoparticles (MSNs) constitute a promising platform for curcumin delivery, mitigating its pharmacokinetic limitations and enabling controlled release at the target site. The incorporation of metals may further enhance the therapeutic effects of curcumin. In this study, a series of curcumin carriers based on MSNs decorated with silver or copper and aminopropyl groups was produced. The resulting nanomaterials contained CuO and AgBr crystallites and exhibited uniform mesoporosity, large pore volumes, and specific surface areas. These materials were evaluated for curcumin delivery, cytotoxicity toward normal and cancer cells of intestinal origin, and antimicrobial activity against a wide range of bacteria and fungi. The most favorable properties were exhibited by the carrier containing AgBr and aminopropyl groups, which showed a selectivity index of 3.8 for cancer cells relative to healthy cells, the highest anticancer activity (IC_50_ = 19.72 µg/mL), and a strong antimicrobial effect against *Pseudomonas aeruginosa*.

## 1. Introduction

Nanotechnology has recently revolutionized medicine, enabling advanced drug delivery, diagnostics, and therapies. Among nanocarriers, mesoporous silica nanoparticles (MSNs) are particularly promising for the encapsulation and targeted delivery of bioactive molecules, especially those with poor solubility or stability [1,2,3,4].

Curcumin (CUR), a natural polyphenolic compound extracted from *Curcuma longa*, is well recognized for its potent anti-inflammatory, antioxidant, and anticancer activities [5,6]. Its anticancer effects are mediated through multiple mechanisms, including the modulation of intracellular signaling pathways that promote apoptosis. CUR also exhibits antimicrobial properties by disrupting bacterial membrane integrity, inhibiting DNA replication, interfering with quorum-sensing mechanisms, and preventing biofilm formation [7]. Despite these beneficial properties, the poor solubility, rapid clearance, and low stability of CUR limit its clinical use. Encapsulation in silica-based nanocarriers can enhance CUR bioavailability, protect it against degradation, and enable targeted and sustained release [1].

To improve CUR loading efficiency, various chemical modifications of the MSN surface have been developed [1,8,9]. Surface modifications can also contribute to increased biocompatibility, controlled drug release, enhanced therapeutic performance, and reduced systemic toxicity [1,8,9,10,11]. Functionalization of MSNs with amine groups has demonstrated particularly promising effects [1]. The incorporation of polyamine ligands onto the MSN surface can significantly enhance drug loading capacity, primarily through electrostatic interactions between the amine groups of the carrier and the polar functional groups of CUR [8]. Amine-functionalized MSNs have emerged as a promising platform for the safe and effective delivery of chemotherapeutic agents, offering superior drug loading efficiency and a controlled, sustained drug release profile compared to unmodified MSNs [12].

A promising strategy involves the use of MSNs for the co-delivery of CUR alongside other bioactive agents to enhance their combined or even synergistic effects [13]. The integration of metals, including silver or copper, into silica–CUR systems enables the development of multifunctional nanotherapeutics that combine drug delivery with antimicrobial and anticancer activities [1,14]. Such nanoplatforms have been proposed for applications in infected tissues to accelerate the healing process, where antimicrobials must be delivered at low doses and with high specificity to enhance therapeutic efficacy while minimizing adverse effects on healthy tissue [13]. Metals, including silver and copper, additionally exert intrinsic anticancer activity by inducing apoptosis, inhibiting cancer cell migration and angiogenesis, potentially reducing metastatic spread.

Despite their numerous advantages, MSNs, particularly those incorporating metal components, pose several toxicological concerns. While unmodified MSNs generally exhibit low acute toxicity, specific surface modifications or the nature of the loaded cargo can contribute to adverse biological effects, including oxidative stress, protein aggregation, and pro-inflammatory responses. Therefore, the design of MSN-based nanocarriers must carefully address potential off-target cytotoxicity, immunogenicity, and unfavorable biodistribution.

The aim of this study was to develop a method for synthesizing optimized silica-based nanomaterials functionalized with immobilized aminopropyl groups and decorated with metal compounds (silver bromide or copper oxide), with the goal of identifying the most effective candidate for the adsorption, transport, and targeted release of CUR. To the best of our knowledge, this is the first study to report silica nanocarriers simultaneously incorporating aminopropyl functionalities and surface-decorated with silver bromide for the controlled delivery of CUR. We evaluated their interactions with normal epithelial cells CCD841CoN and cancer cells Caco-2 as models for investigating selectivity between healthy and cancer cells. Additionally, to evaluate the potential of the obtained materials, their general antioxidant capacity and prospective antimicrobial activity against a broad spectrum of Gram-positive, Gram-negative bacteria and yeasts were assessed. This comprehensive approach enabled the selection of the most promising nanomaterial candidate for potential therapeutic applications in cancer, chronic inflammation, and infections, while prioritizing a simple method of synthesis.

## 2. Materials and Methods

Tetraethyl orthosilicate (TEOS), silver nitrate, copper(II) nitrate trihydrate, curcumin (CUR) and 3-aminopropyl triethoxysilane (APTES) were supplied from Sigma-Aldrich (St. Louis, MO, USA). The porosity template was cethyltrimethylammonium bromide (CTAB) purchased from Acros Organics (Boston, MA, USA). Ethanol (96%) and 25% ammonia were obtained from POCH S.A. (Gliwice, Poland). The phosphate-buffered saline (PBS) was supplied from Merck (Darmstadt, Germany).

### 2.1. Preparation of Nanoparticles

Mesoporous silica materials of MCM-41 type, decorated with silver or copper, were obtained in a short time by templating. Metals were incorporated at the precipitation stage, according to the previously described procedure, with minor modifications. Briefly, 4.4 g of CTAB was dissolved in 250 mL of distilled water [15]. Then 21 mL of 25% ammonia and 1 g of a corresponding metal salt (silver nitrate or copper nitrate trihydrate) were added with vigorous stirring. Once the salt was dissolved, 20 mL of TEOS was added dropwise and the final mixture was stirred continuously for 60 min. All precipitated samples were filtered, washed, dried at 90 °C for 2 h, calcined at a heating rate of 1°/min, and stored at 500 °C for 3 h. Half of the quantities of materials obtained were modified by binding aminopropyl groups.

A quantity of 300 mg of calcined mesoporous silica material was suspended in 25 mL of ethanol and sonicated during 1 min. Next 0.5 mL of APTES was added and the mixture was sonicated during 10 min. and shaken for 2 h. The reaction solution was filtered, and the deposited gel was washed with ethanol and deionized water. The obtained white solid was dried during 24 h at room temperature and next 30 min. at 80 °C.

The following denotations were used for the obtained materials: AgMS or CuMS for the metal-loaded mesoporous silicates containing silver or copper, respectively. Samples with incorporated aminopropyl groups were indicated with Ap letters (ApCuMS, ApAgMS).

### 2.2. Adsorption of Curcumin

A 100 mg portion of material was added to 25 mL of a CUR solution in ethanol (50 mg/L), and the mixture was shaken for 2 h. The slurry was then filtered, and the solid residue was dried under vacuum for 24 h at room temperature. The equilibrium CUR concentration in post-adsorption solution was determined using a UV-VIS spectrophotometer. Materials with adsorbed curcumin are designated by the abbreviation “CUR” at the beginning of the sample label (CUR-CuMS, CUR-AgMS, CUR-ApCuMS, CUR-ApAgMS).

The CUR adsorption efficiency was calculated according to the following equation:

% CUR = 100% × (C_0_ − C_1_)/C_0_ (1)

where C_0_ (mg/L) and C_1_ (mg/L) are the concentrations of CUR at the initial and equilibrium state.

### 2.3. Curcumin Release

A total of 49 mL of buffer solution was mixed with 1 mL of ethanol. Then, 15 mg of the investigated sample was added to 5 mL of this mixture, and the suspension shaken for 48 h at 37 °C. UV-VIS measurements were performed after 24 h and 48 h at a wavelength of 428 nm. Each sample was tested in duplicate.

### 2.4. Curcumin Stability

CUR stability was evaluated in phosphate-buffered saline solutions at pH 7.3 and 5.4. The stock solution was prepared by dissolving 6.25 mg of CUR in 10 mL of ethanol. Subsequently, 1 mL of the stock solution was added to 49 mL of buffer solution, and the mixture was shaken for 2 h at 37 °C. UV-VIS measurements were performed every 10 min at a wavelength of 428 nm.

### 2.5. Sample Characterization

The obtained samples were characterized by nitrogen adsorption–desorption isotherms at 77 K using an ASAP 2405N analyser (Micromeritics, Norcross, GA, USA). The adsorption data were used to determine the specific surface area (S) of the investigated samples using the standard BET method and the total pore volume (V_p_) from the amount of nitrogen adsorbed at a relative pressure of *p*/*p*_0_ = 0.99. The mesopore diameter (D_p_^meso^) was determined from the maximum of the pore size distribution. The crystalline structure of samples was examined by X-ray powder diffraction using an HZG-4 diffractometer (Carl Zeiss, Jena, Germany) with monochromatic CuKα radiation. The diffraction patterns were recorded with a step size of 0.02°.

The quantitative Ag and Cu contents were determined by flame atomic absorption spectrometry (FAAS) using a ZA3300 Atomic Absorption Spectrometer (Hitachi, Japan). The samples were digested in a microwave system (Multiwave 5000, Anton Paar) using HF and HNO_3_. For AgBr-containing samples, the AgBr precipitate was reduced to metallic silver using D-glucose in an alkaline medium and subsequently dissolved in concentrated HNO_3_. Hydrodynamic particle size measurements were performed using Zetasizer Nano ZS ZEN3600 (Malvern Instruments, UK). Approximately 1 mg of each material was suspended separately in 1.2 mL of deionized water, treated with ultrasound for 3 min, and then introduced into the instrument. Zeta-potential measurements were performed using the same instrument in filtered (0.22 µm) PBS. All dynamic light scattering (DLS) measurements were conducted at 25 °C and a scattering angle of 173°. TEM images were obtained using a Talos F200X transmission electron microscope (Thermo Scientific™). The samples were prepared by dropping the ethanol particle dispersion onto a carbon film supported on copper or nickel grids. FTIR spectra were recorded at room temperature using a Nicolet 6700 spectrometer (Thermo Scientific™, Waltham, MA USA) equipped with a Smart iTR diamond ATR accessory, in the range of 500–4000 cm^−1^ at a resolution of 4 cm^−1^. Thermogravimetric analysis (TGA) was performed using a Q-1500 D derivatograph (MOM, Budapest, Hungary). The samples were heated in air from 30 °C to 700 °C at a heating rate of 10°/min. UV-VIS measurements were performed using a UV-1800 spectrometer (Shimadzu, Kyoto, Japan) at a wavelength of 428 nm.

### 2.6. Cells and Culture Conditions

Dulbecco’s Modified Eagle’s Medium (DMEM) with high glucose and phenol red, non-essential amino acids (NEAA), foetal bovine serum (FBS), penicillin/streptomycin solution (P/S), 0.25% (*w*/*v*) trypsin–0.53 mM EDTA, and phosphate-buffered saline (PBS) with or without Ca^2+^/Mg^2+^ were obtained from Merck KGaA (Darmstadt, Germany, and/or its affiliates).

The CCD841CoN cell line (epithelial cells derived from normal human colon tissue) was obtained from the American Type Culture Collection (ATCC; Cat. No. CRL-1790). The Caco-2 cell line (human colon adenocarcinoma cells) was obtained from the European Collection of Authenticated Cell Cultures (ECACC; Cat. No. 86010202). The cells were cultured in DMEM supplemented with 10% FBS, 1% NEAA, and 1% P/S (sDMEM) at 37 °C in a humidified atmosphere containing 5% CO_2_. The cells were routinely tested for mycoplasma contamination using the MycoAlert Mycoplasma Detection Kit (Lonza Walkersville, Inc.).

### 2.7. Cytotoxicity Assays

The cytotoxicity of the generated nanocarriers (CuMS, ApCuMS, CUR-CuMS, CUR-ApCuMS, AgMS, ApAgMS, CUR-AgMS, CUR-ApAgMS) and pure CUR was tested with tetrazolium salt (XTT) reduction by mitochondrial succinate dehydrogenases using the In Cytotox-XTT Cytotoxicity Kit (Xenometrix AG, Allschwil, Switzerland).

Stock suspensions of nanomaterials at 20 mg/mL were prepared in sterile distilled water. The working suspensions were subsequently prepared in sDMEM. The cells at 1 × 10^5^ per 1 mL were seeded in 96-well plates at the volume of 200 µL and left for 24 h to adhere. Thereafter, the medium was removed, and the cells were treated with tested materials at: 800–25 µg/mL for CuMS, CUR-MS and CUR-ApMS, 400–12.5 µg/mL for ApCuMS, 200–6.25 µg/mL for AgMS, ApAgMS, CUR-ApMS, CUR-ApAgMS. The concentrations used in the experiment were determined on the basis of preliminary studies. Stock solution of CUR (25 mg/mL) was prepared in DMSO. To calculate IC50 value of CUR, serial two-fold dilutions of CUR ranging from 200 to 0.78 µg/mL were tested. Cells cultured in medium alone or, in the case of CUR, in medium supplemented with 0.5% DMSO were used as negative controls. 0.1% Triton X-100 (ThermoFisher Scientific, USA) was used as positive controls.

After 48 h of exposure, the medium was collected from MSN-containing samples for subsequent IL-1β and TNF-α analyses. Fresh sDMEM supplemented with a mixture of XTT II and XTT I reagents (2,3-bis[2-methoxy-4-nitro-5-sulfophenyl]-2H-tetrazolium-5-carboxanilide inner salt and buffer) in a 1:100 ratio was then added. The samples were incubated for 3 h, gently mixed, and the absorbance was measured at 480 nm with a reference wavelength of 690 nm using a FLUOstar Omega multi-mode plate reader (BMG Labtech, Germany).

Experiments were performed with at least two independent repetitions of the entire procedure. Each determination was conducted in triplicate. The 50% inhibitory concentration (IC_50_) was determined from the concentration–response curves using a four-parameter logistic regression model. The IC_50_ selectivity index (SI) was calculated using the following formula:

SI = IC_50_ (normal cell line)/IC_50_ (cancer cell line) (2)

where 1 ≤ SI ≤ 3 was considered to indicate lack/low selectivity and SI > 3 indicated high selectivity [16].

The release of two proinflammatory cytokines, IL-1β and TNF-α, as markers of cell irritation was examined using the Human IL-1β and Human TNF-α ELISA Kits (ELK Biotechnology CO., Ltd., Denver, CO, USA). Every sample was tested in duplicate. Tests were performed according to the manufacturer’s instruction. Cytokine levels in the studied samples were calculated using the equation obtained from the standard curve prepared using standard solutions of IL-1β or TNFα. Detection range was 15.63–1000 pg/mL for both cytokines.

### 2.8. Antioxidant Activity Assay

The radical scavenging activity of the studied materials was determined by using DPPH assay. DPPH (2,2-diphenyl-1-picrylhydrazyl) was obtained from Sigma-Aldrich (St. Louis, MO, USA) and dissolved in ethanol to obtain 0.1 mM solution. Ascorbic acid (Sigma-Aldrich, St. Louis, MO, USA, 0.5 mg/mL) was used as reference. Tested samples were CUR at 5 μg/mL and nanomaterials (CUR-CuMS, CUR-ApCuMS, CUR-AgMS, CUR-ApAgMS) at 100 µg/mL prepared in DPPH solution. The reaction mixtures were incubated in dark conditions at room temperature for 30 min. Then, 200 µL of each tested sample was transferred in triplicate to a 96-well titration plate, taking care to avoid transferring the nanoparticles that had settled at the bottom of the reaction vessels. The DPPH solution alone was used as the control. Absorbance was measured at 517 nm using the multi-mode plate reader. The percentage of radical scavenging activity (% RSA) of the samples was calculated according to the following formula:

% RSA = 100% × (OD_517_ control − OD_517_ sample)/Abs control (3)

where RSA is the Radical Scavenging Activity; OD_517_ control is the absorbance of DPPH solution alone at 517 nm; and OD_517_ sample is the absorbance of the DPPH solution with tested samples.

### 2.9. Antimicrobial Activity Assays

The minimal inhibitory concentration (MIC) and the minimum bactericidal/fungicidal concentration (MBC/MFC) of the tested materials were evaluated for antibacterial and antifungal activity using the microdilution broth method in Mueller-Hinton broth (Biomaxima, Lublin, Poland) or RPMI 1640 (with L-glutamine and a pH indicator but without bicarbonate) supplemented with glucose to a final concentration of 2% (RPMI 2% G) and MOPS (3-(N-morpholino) propanesulfonic acid (Merck, Darmstadt, Germany and its affiliates) for growth of bacteria and fungi, respectively. The panel of reference microorganisms, including Gram-positive bacteria (*Staphylococcus aureus* ATCC 25923, *Staphylococcus aureus* ATCC BAA-1707, *Staphylococcus epidermidis* ATCC 12228, *Micrococcus luteus* ATCC 10240, *Enterococcus faecalis* ATCC 29212, *Bacillus cereus* ATCC 10876), Gram-negative bacteria (*Escherichia coli* ATCC 25922, *Salmonella Typhimurium* ATCC 14028, *Klebsiella pneumoniae* ATCC 13883, *Pseudomonas aeruginosa* ATCC 9027, *Proteus mirabilis* ATCC 12453), and fungi (*Candida albicans* ATCC 10231, *Candida parapsilosis* ATCC 22019, *Candida glabrata* ATCC 90030) was investigated. The sterile 96-well polystyrene microtitration plates (Nunc, Roskilde, Denmark) were prepared by dispensing 100 μL of an appropriate dilution of the tested compounds in broth medium or per well by serial two-fold dilutions to obtain final concentrations of the tested compounds ranging from 1000 to 7.8 µg/mL. The inocula were prepared with fresh microbial cultures in sterile 0.85% NaCl to match the turbidity of 0.5 McFarland standard and were added to wells to obtain a final density of 5 × 10^5^ CFU/mL for bacteria and 5 × 10^4^ CFU/mL for yeasts (CFU, colony forming units). After incubation (35 °C for 24 h), the MICs were assessed visually and spectrophotometrically at 600 nm as the lowest concentration of the compounds that shows complete growth inhibition of the reference microbial strains.

Appropriate DMSO control (at a final concentration of 10%), a strain growth control (inoculum without the tested materials), and a negative control (the tested materials ranged from 1000 to 7.8 µg/mL without inoculum) were included on each microplate. MBC or MFC was obtained by culturing 5 µL from each well that showed growth inhibition, from the last positive one, and from the growth control onto recommended agar plates. The plates were incubated at 35 °C for 24 h for all microorganisms. The MBC/MFC was defined as the minimum concentration of compounds that kills 99.9% of the test microorganisms in the original inoculum. The MIC was determined based on turbidity, evaluated both visually and by spectrophotometric measurement using absorbance microplate reader BioTek ELx800 (BioTek^®^ Instruments, Inc., Winooski, VT, USA) at 600 nm. All tests were performed in triplicate, with the highest recorded value reported.

## 3. Results

### 3.1. Physicochemical Characteristics

**Figure 1:**
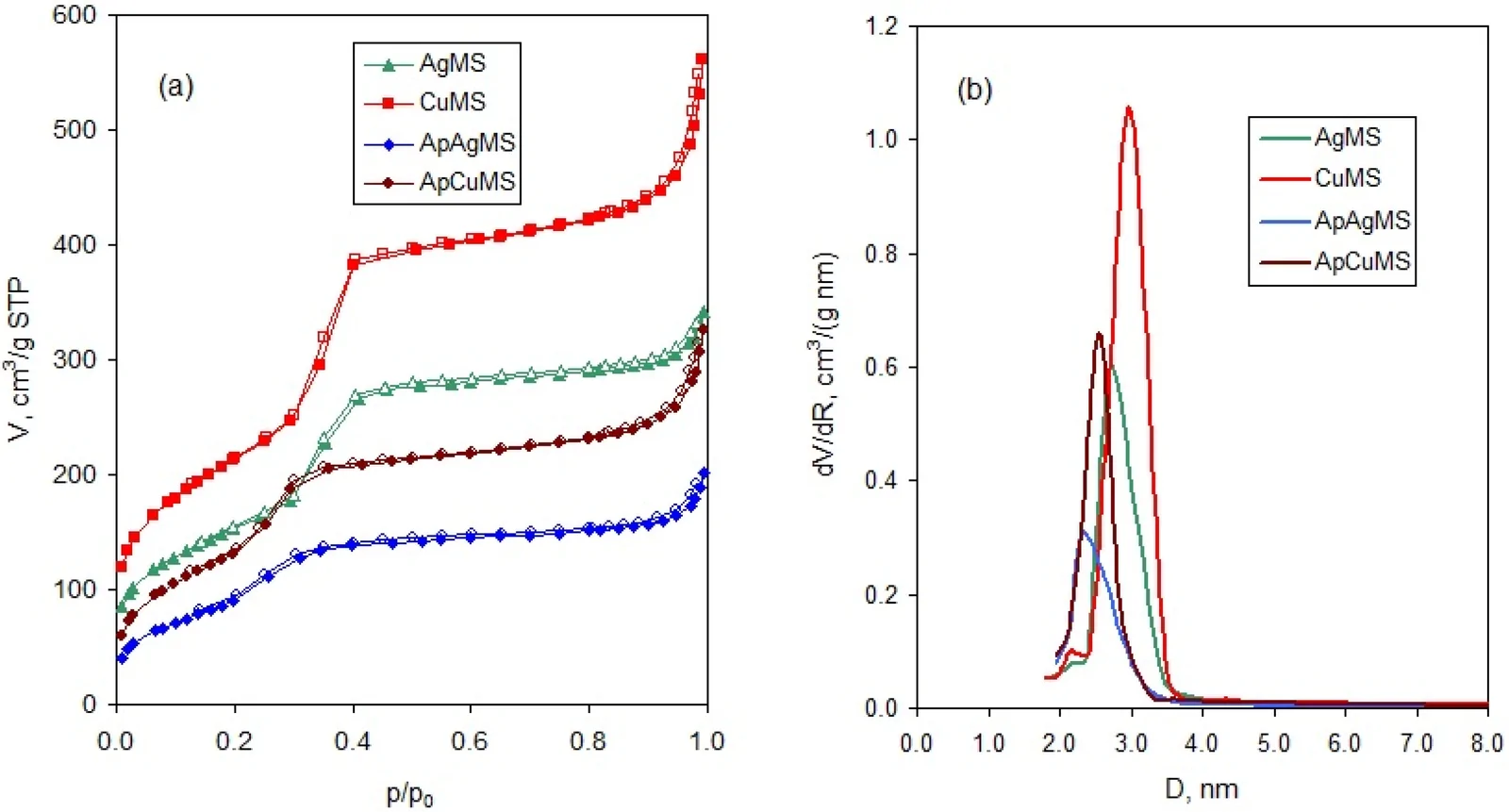
Nitrogen adsorption isotherms (**a**) and pore size distributions (**b**) of investigated samples.

The structural parameters of the obtained materials were characterized using low-temperature nitrogen adsorption/desorption measurements. All the isotherms (Figure 1a) were classified as type IV according to the IUPAC convention [17] and were typical for mesoporous materials. The narrow hysteresis loops observed in the isotherms were classified as H4-type and suggested the presence of cylindrical, slit-like mesopores. The sharp inflection in the relative pressure range of *p*/*p*_0_ = 0.2–0.4 corresponded to capillary condensation within relatively uniform mesopores. The mesopore diameters ranged from 2.3 to 3.0 nm, which is suitable for CUR adsorption. The nitrogen adsorption capacity, as well as the pore dimensions, decreased considerably following the introduction of aminopropyl groups (Table 1).

**Figure 2:**
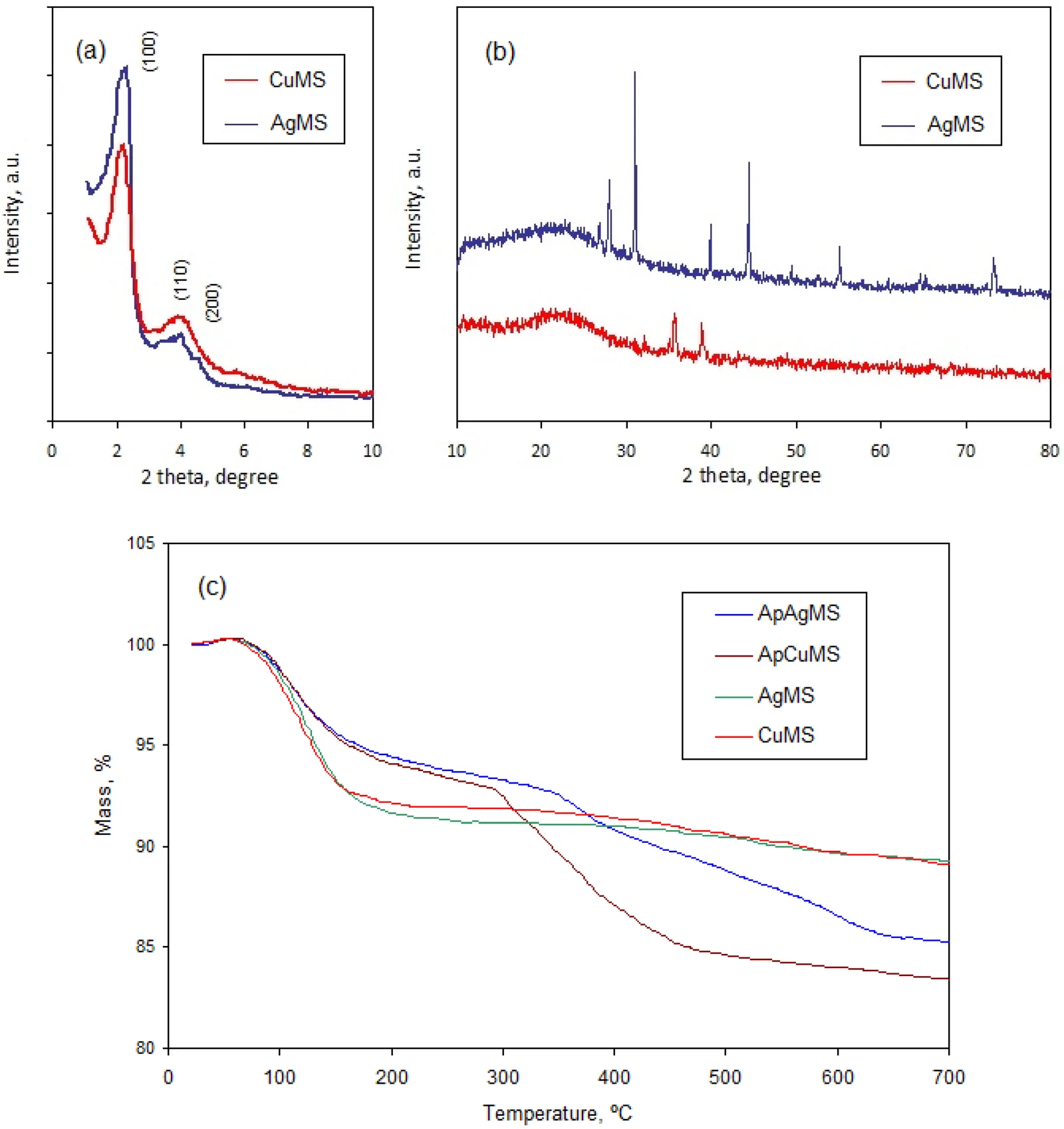
X-ray diffraction patterns in (**a**) small and (**b**) wide 2 theta range, and (**c**) thermogravimetry analysis of investigated samples.

The crystallographic structure of the obtained materials was examined using X-ray diffraction method. The XRD patterns of parent samples decorated with metal compounds are shown in Figure 2. In the small-angle region (Figure 2a), all materials exhibited peaks characteristic of 2D long-range ordering with hexagonal symmetry, with visible re-flections indexed as (100), (110) and (200), characteristic of mesoporous MCM-41 type silicates. In the wide-angle region (Figure 2b), a broad peak at approximately 2θ = 22° was observed, confirming the amorphous character of the silica walls.

The strong reflections observed in the XRD pattern of the AgMS sample at 26.7°, 31.0°, 44.3°, 55.0°, and 64.5° were characteristic of AgBr, as indexed in the PDF#06-0438 database. At 32.6°, 35.7° and 38.9° for CuMS material appear diffraction peaks attributed to the CuO crystallites (copper oxide standard PDF#48-1548). The CuO particles were formed during the annealing of samples prepared using copper nitrate at 550 °C, during which Cu^2+^ ions reacted with oxygen from the air. Silver bromide was formed during the precipitation stage. The surfactant CTAB, which acts as a porosity template and contains Br^−^ ions, reacted in solution with Ag^+^ ions originating from AgNO_3_, resulting in the formation of a poorly soluble AgBr precipitate. Both CuO and AgBr were thermally stable up to 700 °C.

The quantitative content of silver and copper deposited on the silica support in the form of AgBr and CuO, respectively, was determined by flame atomic absorption spectrometry (FAAS). The copper content was 32.5 mg per gram of carrier, while the silver content was 44.75 mg per gram of carrier. These values corresponded to 0.51 mmol of copper and 0.42 mmol of silver, respectively. The initial amounts of the substances used for sample synthesis had been selected in advance to obtain similar molar quantities of the metals in the resulting products.

The amount of immobilized aminopropyl groups was determined using thermogravimetric (TG) analysis. The TG analysis results are presented in Figure 2c. For all prepared materials, the first weight loss, occurring below 150 °C, was attributed to the desorption of physisorbed water. The highest water content was observed for the CuMS and AgMS samples, which exhibited weight losses of 8% and 8.5%, respectively. Samples containing aminopropyl groups exhibited approximately 6% (*w*/*w*) weight loss due to adsorbed water, which may be attributed to their more hydrophobic character and lower pore volumes. The next step, occurring between 300 and 650 °C, was attributed to the thermal degradation of the organic groups. A higher amount of aminopropyl groups was desorbed from the copper-containing sample (8.5%), compared with the silver-containing sample (7%). The decomposition of the aminopropyl groups was considerably faster in the ApCuMS material. This process began at approximately 300 °C, and almost all groups were decomposed by 500 °C. This observation may indicate a catalytic effect of CuO on the thermal decomposition of the aminopropyl chains.

**Figure 3:**
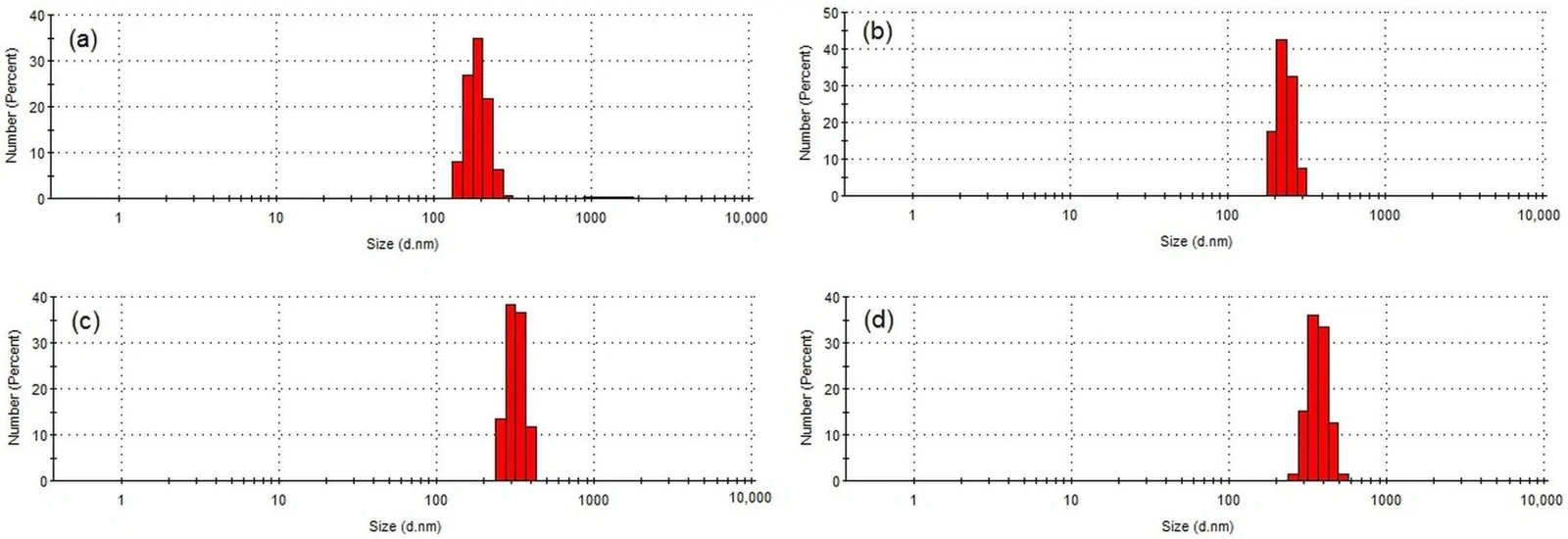
DLS size distributions of: (**a**) AgMS, (**b**) CuMS, (**c**) ApAgMS, (**d**) ApCuMS samples.

DLS measurements were performed to examine the zeta potential and particle size of the obtained materials. The DLS particle-size distributions by number are presented in Figure 3.

The histograms obtained were relatively narrow. The average hydrodynamic diameters of particles, determined from maxima in the DLS-size distributions, were 190 nm, 295 nm, 220 nm and 342 nm for the AgMS, ApAgMS, CuMS, and ApCuMS samples, respectively. The PDI values of the obtained materials ranged from 0.381 to 0.533. The zeta potential, which provides information on the electrostatic interactions between nanoparticles and is commonly used to characterise surface charge and assess colloidal stability, was also determined. The zeta potential values measured for the AgMS, ApAgMS, CuMS, and ApCuMS materials were −36.9 ± 0.058 mV, 33.4 ± 0.757 mV, −37.1 ± 1.130 mV and 30.7 ± 0.839 mV, respectively.

**Figure 4:**
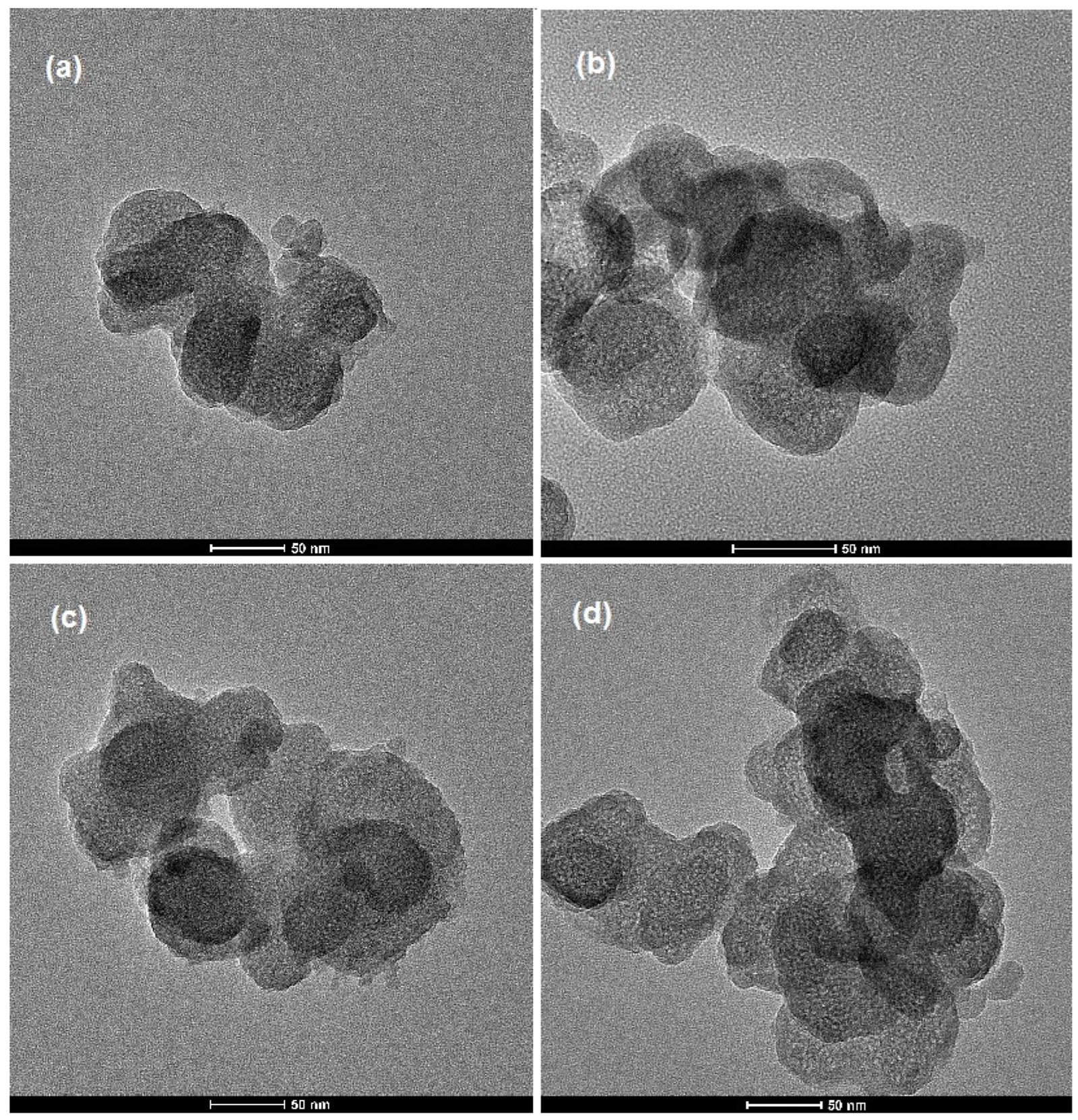
TEM images of: (**a**) AgMS, (**b**) ApAgMS, (**c**) CuMS, (**d**) ApCuMS samples.

To examine the morphology and size of the obtained nanoparticles, TEM was employed. The aggregates visible in the TEM images (Figure 4) consist of nearly spherical particles with a mesoporous network characteristic of MCM-41-type materials. However, their sizes appear to be smaller than those determined by the DLS method.

**Figure 5:**
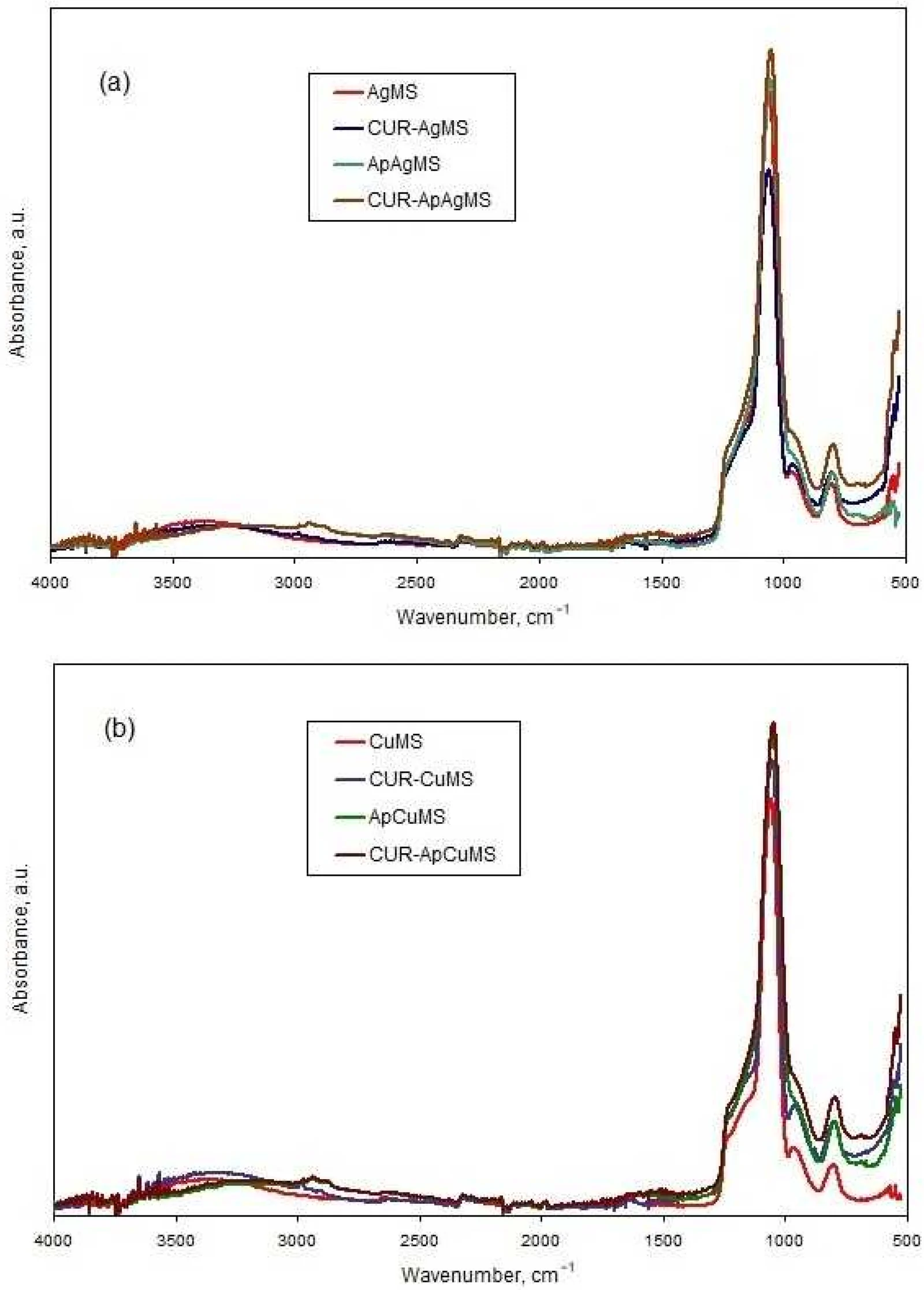
FT-IR spectra of samples containing (**a**) AgBr and (**b**) CuO.

To identify the functional groups and investigate potential interactions between them, the FT-IR spectroscopy was used. The FT-IR spectra of the investigated samples and pure CUR are presented in Figure 5 and Supplementary Figure S1 (available online).

The FT-IR spectra of the MS samples decorated with CuO and AgBr (Figure 5) exhibited bands characteristic of silica-based materials, including a strong band at 1070 cm^−1^ corresponding to the asymmetric stretching vibration of Si–O–Si and a band at 811 cm^−1^ assigned to the symmetric stretching vibration of Si–O–Si. The band observed at 975 cm^−1^ was attributed to the stretching vibration of Si–OH groups and decreased in intensity following aminopropyl immobilization, likely due to the consumption of silanol groups during the modification process. Furthermore, the broad band in the range of 3000–3600 cm^−1^ shifted toward lower wavenumbers. The presence of aminopropyl groups was further confirmed by the C–H stretching vibrations of the propyl chain at 2950 and 2880 cm^−1^.

Following CUR adsorption, several new signals appeared in the spectra. These signals were relatively weak because the amount of CUR was substantially lower than that of the carrier in the resulting nanostructure. In the 1400–1600 cm^−1^ region, a broad, relatively flat band was observed, which was attributed to C–C stretching vibrations within the aromatic rings of CUR. A small, distinct band at 2990 cm^−1^ was assigned to C–H stretching vibrations of the –CH_3_ group. The rapidly increasing spectral intensity near 500 cm^−1^ was attributed to vibrations associated with the methoxy group, which exhibited a signal at approximately 450 cm^−1^.

The CUR adsorption efficiencies, determined using UV–Vis spectrophotometry and calculated according to Equation 1, were 78.5%, 80.4%, 81.8%, and 82.5% for CUR-AgMS, CUR-CuMS, CUR-ApAgMS, and CUR-ApCuMS, respectively. These values corresponded to CUR loading capacities (expressed in mg CUR per gram of carrier) of 9.81, 10.05, 10.23 and 10.32, respectively, for CUR-AgMS, CUR-CuMS, CUR-ApAgMS and CUR-ApCuMS.

### 3.2. Curcumin Release

**Figure 6:**
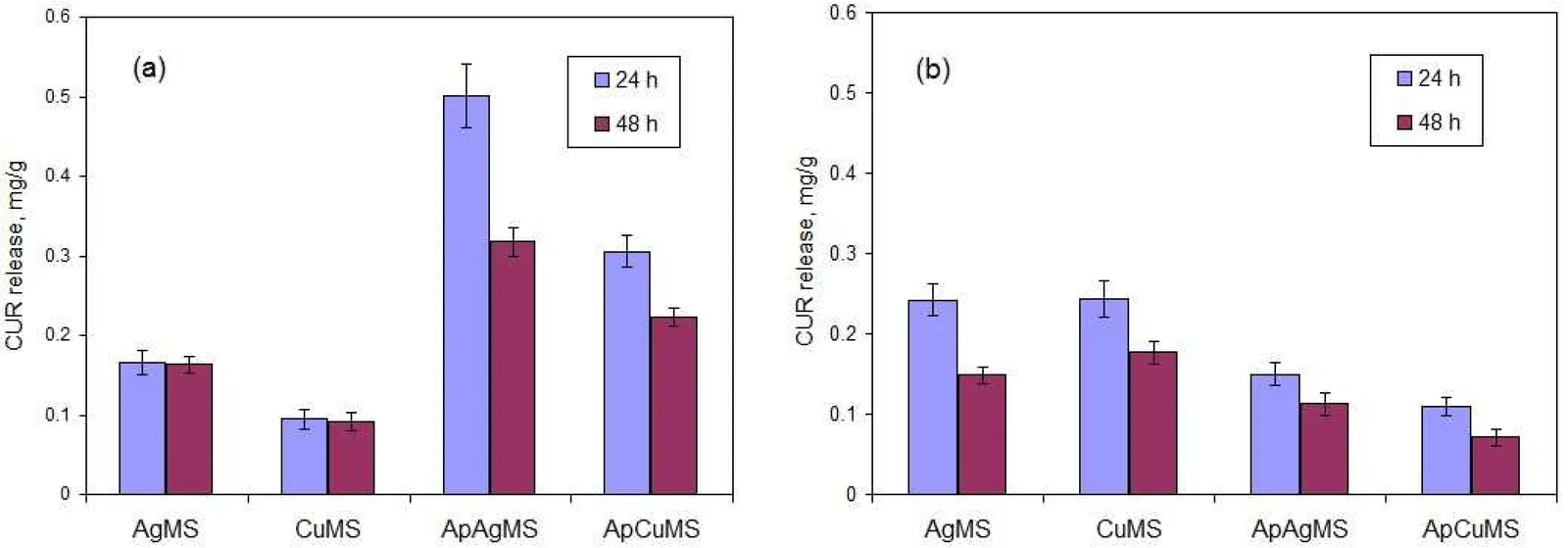
CUR release from the investigated carriers at (**a**) pH 5.4 and (**b**) pH 7.3 after 24 and 48 h.

CUR release study was performed at neutral (pH 7.3) and acidic (pH 5.4) conditions. The results were expressed as the CUR amount measured in solution after 24 and 48 h of release from 1 g of carrier-adsorbate system. These two time periods were chosen to allow for a more accurate comparison of the effect of individual carriers on the CUR desorption process. Furthermore, they correspond to the duration of the biological experiments conducted as a part of this study. In aqueous solutions, CUR decomposes relatively quickly. A determined half-life of pure CUR in the phosphate-buffered saline solutions at pH 5.4 and 7.3, containing 2% ethanol addition, at 37 °C was about 80 min. and 60 min., respectively—see Figure S2 in the Supplementary Materials (online). The measured drug concentration is therefore significantly lower than that which actually desorbed from the carrier. For this reason, it seems better to use and discuss the actual amount of the active form of the drug present in the reaction solution rather than its percentage release from the adsorbent. Obtained data, collected in Figure 6, show the preference for CUR desorption in acidic media from samples decorated with amino groups, whereas at pH 7.3 these samples exhibit much lower desorption. In contrast, samples lacking aminopropyl groups demonstrated the opposite behavior, although the difference was less pronounced than in the presence of Ap groups.

### 3.3. Cytotoxic and Antioxidant Activity

The concentrations of the tested materials were selected based on preliminary studies indicating higher cytotoxicity of nanocarriers containing silver bromide (AgBr). In addition, MSNs functionalized with Ap groups exhibited greater cytotoxicity at higher concentrations than their non-functionalized counterparts, highlighting the influence of surface modification on biological responses.

The cytotoxicity test was performed by a standard method using the XTT dye, which is converted into a formazan product by metabolically active cells, serving as a measure of cell viability and proliferation. Although there are some concerns regarding the use of tetrazolium salt based assays, particularly MTT, due to the possibility that silica nanoparticles may stimulate exocytosis of formazan crystals and thus lead to overestimation of the cytotoxicity of these NPs [18], the XTT assay results observed in our study correlated well with the level of the cell culture confluence and other microscopic changes observed simultaneously.

The tested nanomaterials exhibited variable effects, which depended on both their composition, primarily the presence of CuO or AgBr, and the type of cells exposed (normal or cancer cells). Overall, carriers containing AgBr exhibited higher toxicity, as reflected in the calculated IC_50_ values. Simultaneously, the IC_50_ data indicate greater sensitivity of cancer cells, particularly for materials containing AgBr (Table 2). The presence of Ap groups also had a noticeable effect, increasing cytotoxicity compared to the corresponding materials lacking these groups. Concentration-dependent responses of the cells are given on Supplementary Figure S3 (available online) to better illustrate the cell response to the tested NPs.

Although the strong cytotoxic effect of AgBr-containing NPs is evident, particularly against Caco-2 cells, the presence of CUR appears to attenuate this effect (except when combined with Ap groups).

We also assessed the release of the pro-inflammatory cytokines IL-1β and TNF-α by the exposed cells, with a detection limit of 15.63 pg/mL for both cytokines. The observed responses depended on the type of nanomaterial, its concentration, and the cell type. In the culture medium of Caco-2 and CCD841CoN cells cultured in the absence of NPs, neither cytokine was detected. Cu-containing nanomaterials did not induce the release of either cytokine by CCD841CoN cells, even at the highest concentrations tested. In Caco-2 cells exposed to Cu-containing materials, IL-1β levels remained below the detection limit regardless of NP concentration. Similarly, TNF-α levels did not increase significantly, except for a slight elevation in cells incubated with ApCuMS at concentrations that were cytotoxic according to the XTT assay (100 and 200 μg/mL). Nevertheless, TNF-α concentrations remained low, ranging from approximately 18 to 22 pg/mL, only slightly above the detection limit.

Caco-2 cells exhibited increased TNF-α release following exposure to AgBr-decorated NPs (Table 2b). This increase was not accompanied by a significant release of IL-1β, which was detectable only in cells treated with ApAgMS at 50 μg/mL (15.7 ± 0.001 pg/mL). Similarly, CUR-ApAgMS at the highest tested concentration induced IL-1β release at a comparable, near-detection-level concentration (15.6 ± 0.015 pg/mL). These results indicate that TNF-α may serve as a sensitive and reliable marker of the cytotoxic effects of the tested MSNs in Caco-2 cells.

The antioxidant activity of the obtained nanomaterials was tested by measuring their ability to quench the DPPH free radical by donating a hydrogen atom, resulting in a change in the colour of the solution. This study confirmed the strong antioxidant activity of CUR, reaching nearly 76% RSA at 5 µg/mL of the compound (Table 3). The DPPH-to-DPPH-H reduction activity was demonstrated also by all tested CUR-containing nanocarriers, at a range of approx. 41–49% RSA, which is a satisfactory effect considering the amount of CUR incorporated into the tested 100 µg/mL nanocarrier suspensions.

### 3.4. Antimicrobial Activity

Antimicrobial activities of the studied materials were tested against a wide panel of Gram-negative bacteria, Gram-positive bacteria, and yeasts (Table 4). CUR showed a mild or moderate activity against Gram-positive reference bacterial strains of *S. aureus*, *S. epidermidis*, *M. luteus* and *B. cereus* with MIC ranged from 312.5 to 625 µg/mL. Within the tested concentration range (MIC > 1000 µg/mL), CUR exhibited no observable antibacterial activity against the evaluated Gram-negative reference strains. Similarly, Cu-loaded MSNs demonstrated no detectable antimicrobial activity across the tested range of 1000–7.8 µg/mL. On the contrary, AgMS revealed good bioactivity towards all tested reference bacterial and fungal strains (MIC ranged from 31.25 to 125 µg/mL). The highest antimicrobial activity was detected for ApAgMS. It was found that all tested Ag-decorated MSNs exhibited bactericidal activity since the MBC/MIC index for most of the tested Gram-positives was <4. Interestingly, MSNs loaded with CUR exhibited slightly reduced or comparable antimicrobial activity against most of the tested microorganisms when compared to both AgMS and ApAgMS. However, CUR-ApAgMS displayed superior efficacy against *S. Typhimurium* based on MIC values, and against *P. aeruginosa* in terms of minimum bactericidal concentration.

## 4. Discussion

To address the challenges of precisely delivering drugs to target sites while maintaining their desired properties and limiting side effects, various nanoparticle-based delivery systems have been developed. However, the most widely used nanocarriers in drug delivery applications are MSNs with their relevant advantages [19]. Silica nanoparticles can be synthesized by various methods, including precipitation, wet chemical synthesis, acid treatment, reverse microemulsion, hydrothermal, and sol–gel techniques. Among these, the sol–gel method is most commonly used, as it allows precise control over particle size and morphology [20,21].

MSNs can be functionalized with a variety of organic groups, enabling interactions with diverse molecules. Many studies used modified MSNs with environment-responsive gatekeepers sensitive for pH, temperature or redox conditions [1]. Amine-functionalized silica nanoparticles, typically prepared using 3-aminopropyltriethoxysilane (APTES), offer controlled release capabilities for different molecules [21]. The strong electrostatic interaction between the carbonyl groups of CUR and the amine groups of APTES-functionalized MSNs facilitates efficient CUR loading.

In this study, MSNs decorated with AgBr and CuO were successfully synthesized using CTAB as a template. Their ordered mesoporous structure was confirmed by nitrogen adsorption method (Figure 1 and Table 1) and X-ray diffractometry (Figure 2a). The structure parameters of the starting samples with metals vary due to the different forms of the compounds obtained. The silver bromide particles are much larger than copper oxide ones and hence occupy a larger pore volume. Furthermore, different degrees of metal compound particle deposition on a pore surface or their incorporation into the pore walls may occur. The existence of these compounds inserted into the synthesized samples was verified by wide-angle XRD (Figure 2b), where the reflexes characteristic for AgBr and CuO are visible. The introduction of aminopropyl groups also considerably reduces the pore dimensions (Table 1); however, they are still suitable for CUR adsorption. Moreover, the presence of aminopropyls does not disturb the hexagonal arrangement of uniform mesopores (Figure 2a). The amount of immobilized Ap groups was determined by the thermogravimetric method (Figure 2c). The lower quantity of Ap bound to the silica material with AgBr deposits may be due to the smaller surface area and pore volume of the silver-containing sample.

It is well established that the optimum size of nanoparticles for biomedical applications is generally within the range of 10 and 300 nm [22]. The materials synthesized in this study appear to fall within this range. Although the average particle sizes determined from the DLS size distributions (Figure 3) are somewhat higher than this range for some samples, it should be noted that DLS measures the hydrodynamic diameter, which may exceed the actual physical diameter of the nanoparticles. The hydrodynamic diameter represents the effective diameter of a particle moving through a fluid and reflects not only the particle core but also the surrounding solvent molecules and associated ions. Thus, DLS measurements provide information on particle size in a liquid environment and are influenced by particle–solvent interactions. Considering the length of the aminopropyl chains, the increase in hydrodynamic diameter observed following Ap functionalization may be attributed, at least in part, to changes in the interactions of the modified particles with the surrounding liquid.

The zeta potential is an important parameter for characterizing nanoparticle surface charge and assessing colloidal stability. Particles exhibiting zeta potential values above +30 mV or below −30 mV are commonly considered to possess relatively high electrostatic stability, owing to strong interparticle repulsion that reduces the tendency to aggregate [23]. Accordingly, the materials synthesized in this study appear to exhibit relatively good colloidal stability. The relatively high positive zeta potential of the Ap-modified samples may promote electrostatic association with negatively charged cancer cells, and it may also increase their affinity for negatively charged serum proteins, such as albumin. These properties should therefore be taken into consideration when evaluating potential administration routes and the biological behaviour of these nanomaterials in future biomedical applications.

The amount of CUR used in the present study was selected based on our previous experiments and a review of the literature, with the aim of achieving a therapeutically relevant effect while avoiding excessive coverage of the carrier surface by the adsorbed active compound, which could mask the intrinsic properties of the nanocarrier. Comparison of the CUR adsorption results obtained in the present study with those reported by other researchers for analogous systems without metal compounds [24] indicates that the presence of both aminopropyl groups and copper oxide or silver bromide increases the efficiency of CUR adsorption compared with bare MCM-41. Moreover, the incorporation of CuO and AgBr further enhanced the amount of adsorbed CUR, particularly during the relatively short adsorption period of 2 h. CUR may interact with silver and copper species through coordination interactions, potentially forming complexes, as suggested by the disappearance of the FT-IR band characteristic of the carbonyl group of CUR (approximately 1630 cm^−1^) (Figure 5).

The attempt of combining CUR with other bioactive substances using modified MSNs has already been explored in various configurations. One interesting approach involved the fabrication of a nanocarrier based on silver-modified mesoporous silica loaded with CUR and capsaicin. The release profiles of the loaded bioactive compounds demonstrated improved solubility at pH 5.5 [25]. Our material differs from the cited system in terms of the surface modification strategy and, consequently, its toxicological profile and biological effects. Nevertheless, similarly to the cited study, we observed enhanced CUR release from the MSNs under acidic conditions. This pH-dependent behaviour is consistent with our aim of designing silica-based nanocarriers capable of delivering and releasing CUR at target sites characterised by a low pH [26,27].

The tumour microenvironment differs markedly from that of normal tissue, both intracellularly and extracellularly. Tumours typically exhibit a more acidic extracellular milieu, while maintaining relatively neutral or slightly alkaline intracellular conditions [28]. This pH gradient provides an opportunity for the controlled release of therapeutic cargo from pH-responsive nanocarriers [29]. In our study, CUR release was evaluated over the pH range of 7.3–5.4, and the desorption rate of CUR from the nanocarrier increased as the pH of the medium decreased (Figure 6). Under acidic conditions, the aminopropyl groups on the MSN surface become protonated, increasing the positive surface charge of the carrier and thereby potentially weakening its interactions with CUR. Conversely, as the pH increases, deprotonation of the amino groups and changes in the ionisation state of CUR may promote stronger interactions between the two components. Hydrogen bonding between the phenolic groups of CUR and the primary amino groups of the carrier may contribute to CUR retention. The spectral data (Figure 5) suggest that, under neutral conditions, CUR interacts with the surface of the amine-functionalised MSNs, potentially involving interactions between the –NH_2_ groups and oxygen-containing functional groups of CUR, including phenolic and enolic groups (Figure S4). These interactions may facilitate CUR retention within the carrier during transport while allowing enhanced release under more acidic conditions.

CUR exhibits a broad range of biological activities, including anticancer and antibacterial effects, which may be further enhanced by the incorporation of metallic NPs [14]. Nanometals are well recognized for their diverse therapeutic applications. Silver has demonstrated anticancer activity through several mechanisms, including the generation of reactive oxygen species (ROS), induction of DNA damage, and disruption of cell-cycle progression, ultimately leading to apoptosis or other forms of cell death [30,31]. Another metal considered in the design of our nanocarriers was copper. CuO nanoparticles have been reported to exhibit notable anticancer activity by inducing apoptosis, cuproptosis, and ferroptosis, partially through mechanisms involving mitochondrial dysfunction, thereby inhibiting tumour growth and potentially causing preferential damage to cancer cells [32,33,34]. Previous studies have also reported that certain metal–CUR complexes exhibit greater biological activity than the corresponding individual compounds [14]. The combination of two biologically active components may therefore offer several advantages, as their activities may complement each other and potentially enhance the overall biological efficacy of the resulting nanomaterial.

In this context, the MSNs produced in our study and functionalised with CuO or AgBr were designed to potentiate the biological effects of CUR. However, enhanced activity attributable to the combined presence of a metal component and CUR was observed exclusively for the amine-functionalised formulation decorated with AgBr and loaded with CUR. This formulation was effective against Caco-2 cancer cells with IC_50_ of 19.72 μg/mL and the SI of 3.8. The high cytotoxicity toward cancer cells, together with a selectivity index indicating preferential activity toward target cells, can be considered particularly favourable. At the same time, IC_50_ of free CUR on Caco-2 cells in our study was 18.26 μg/mL, whereas studies using a similar cell line and exposure time (48 h) reported IC_50_ values in the range of 11–14 µg/mL [35,36]. Considering that the amounts of CUR released from our carriers were low, the observed cytotoxic effect on cancer cells appear to result from the combined activity of the individual nanomaterial components, together with the specific release kinetics of CUR. Additionally, the relatively similar IC_50_ values observed for free CUR and CUR-loaded nanocarriers may be the effect of the limited stability of free CUR and its rapid degradation in the culture medium during cytotoxicity testing. Consequently, a higher nominal concentration of free CUR may be required to achieve a comparable biological effect.

Nanoparticle carriers can increase the aqueous solubility of their cargo and may thereby reduce the total dose required. An important limitation of CUR is its low bioavailability, which is associated with poor absorption and rapid metabolism [1,37,38]. CUR is poorly soluble in water (~11 ng/mL), chemically unstable, susceptible to temperature- and pH-dependent degradation, and rapidly eliminated from the body [1]. In acidic environments, the keto tautomeric form of CUR predominates (Supplementary Figure S4), exhibiting antioxidant activity and increased stability due to resonance stabilisation of the carbon-centred radical. In neutral and basic solutions, the enolic form predominates and is more susceptible to heptadienone chain cleavage and autoxidation. Combining CUR with the nanocarriers developed in this study may help overcome some of these limitations by improving its solubility, stability, and cellular uptake. According to our findings, the release of CUR from the nanocarrier, even under the most favourable acidic conditions, was not rapid but rather prolonged. This sustained release may help maintain the availability of CUR over an extended period by continuously supplying new portions of the compound as previously released CUR undergoes degradation.

Although CUR has been reported to exert a broad range of potential anticancer activities against colorectal adenocarcinoma and other gastrointestinal malignancies [39], the present study was limited to the Caco-2 cell model. Nevertheless, the favourable properties observed for the selected nanocarriers suggest that their biological activity may not be restricted to colorectal cancer. This possibility should be investigated in future studies using a broader panel of cancer cell lines representing different tissue origins. Such studies could help identify tumour types that are particularly susceptible to these nanocarriers and determine whether their activity depends on tumour-specific cellular characteristics.

Cytokine release in cancer and normal cell models was assessed to evaluate the pro-inflammatory response and potential adverse effects of the tested nanomaterials. Elevated cytokine levels, particularly in normal cells, may indicate a potential pro-inflammatory or irritative effect. IL-1β release was detected only in cells exposed to silver-containing nanoparticles, whereas TNF-α showed a distinct response pattern, with markedly elevated levels observed in cancer cells treated with MSNs, particularly those decorated with silver bromide and aminopropyl groups. Comparison of Ap-functionalised MSNs with and without CUR suggested that the presence of CUR slightly attenuated this effect (Table 2b).

As previously reported, CUR exhibits antibacterial activity against both Gram-negative and Gram-positive bacteria [7]. However, in the present study, free CUR showed no detectable antimicrobial activity within the tested concentration range. Theoretically, CUR’s antimicrobial efficacy can be enhanced through interactions with metals such as Cu or Ag. Previous studies have demonstrated that AgBr facilitates greater availability of Ag^+^ ions than elemental silver, and its incorporation into various materials confers antibacterial properties [40,41]. In our study, metal-decorated MSNs also displayed antimicrobial activity, with AgBr-based carriers showing pronounced effects. In contrast, CuO-decorated forms unexpectedly lacked significant activity against either Gram-positive or Gram-negative bacterial strains, as well as the tested yeast strains. The stronger biological effects observed for the AgBr-decorated MSNs compared with their CuO-containing counterparts are very likely related to differences in the mechanisms underlying their biological activity and metal-ion release. Although AgBr is a sparingly soluble compound, AgBr-based nanomaterials have been shown to exhibit cytotoxic and genotoxic effects, as demonstrated in human lymphocytes [42]. The biological activity of AgBr may be related, at least in part, to the release of Ag^+^ ions, which can interact with cellular targets. AgBr nanoparticles have been shown to release measurable amounts of Ag^+^ and provide a sustained source of these ions, thereby exhibiting pronounced biological activity [43]. In AgBr–silica systems, the silica matrix can control the diffusion of Ag^+^ while still maintaining prolonged cytotoxic and antibacterial activity. AgBr-containing silica nanomaterials have already demonstrated strong activity against both Gram-positive and Gram-negative bacteria, with Ag^+^ release proposed as an important contributor to their antibacterial effects [44]. In contrast, in our study the CuO-decorated MSNs exhibited milder effects compared with the silver-containing ones. However, copper compounds also exhibit evident cytotoxic and antibacterial activity [45], primarily through Cu-ion release and associated oxidative and membrane-damaging effects. The magnitude of these effects depends strongly on the amount, dispersion, and physicochemical characteristics of the CuO phase [46]. Therefore, the greater activity of the AgBr-containing carriers observed in the present study may reflect more effective Ag^+^ bioavailability and/or stronger interactions of silver species with bacterial and mammalian cellular targets. However, this interpretation should be considered system-specific, as differences in particle size, metal loading, surface functionalization, dissolution kinetics, and cellular uptake can substantially affect the biological response. Dedicated mechanistic experiments, such as measurements of metal-ion release, intracellular ROS generation, membrane integrity, and cellular uptake, would therefore be required to establish the precise mechanisms responsible for the observed differences.

The properties of the obtained nanocarriers are particularly noteworthy in the context of infections associated with acidic environments. Such infections include, for instance, those caused by *Pseudomonas aeruginosa*, a Gram-negative bacterium resistant to most antibiotics but susceptible to CUR [41]. The effectiveness of Ag NPs against *P. aeruginosa* has been previously demonstrated, suggesting their potential use as an alternative to currently used conventional antimicrobials or to enhance the antimicrobial activity of antibiotics [47]. Bactericidal effects of designed formulation with silver on *P. aeruginosa* were observed in our study.

## 5. Conclusions

This study demonstrates the potential advantages of curcumin-loaded mesoporous silica nanoparticles functionalized with aminopropyl groups and decorated with silver bromide (AgBr) for potential applications in cancer therapy and antibacterial treatment. The developed nanocarrier exhibited pH-responsive CUR release, with enhanced desorption under acidic conditions, which may be advantageous for drug delivery to acidic tumor or infection-associated microenvironments. Importantly, the CUR-loaded AgBr-containing MSN demonstrated selective cytotoxicity toward cancer cells compared with normal cells, as well as notable antibacterial activity. These findings indicate that the combination of mesoporous silica, aminopropyl functionalization, AgBr, and CUR can provide complementary biological and physicochemical properties within a single nanocarrier. Furthermore, the synthesis procedure is relatively simple and cost-effective, supporting the potential for further development of this nanomaterial. Nevertheless, additional studies are required to elucidate the underlying mechanisms of its anticancer and antibacterial effects and to evaluate its stability, safety, and therapeutic efficacy in more complex biological models. Overall, the obtained results highlight CUR-loaded, AgBr-decorated MSNs as a promising multifunctional platform for further investigation in cancer and antimicrobial applications.

## Figures and Tables

**Table 1:** Structure parameters of the investigated samples obtained from nitrogen adsorption data.

Sample	S_BET_, m^2^/g	V_p_, cm^3^/g	D_p_^meso^, nm
AgMS	559	0.53	2.7
CuMS	774	0.87	3.0
ApAgMS	341	0.31	2.3
ApCuMS	492	0.50	2.6

**Table 2:** The IC_50_ of tested nanomaterials for (a) CCD841CoN and Caco-2 cells after 48 h incubation and selectivity index (SI); (b) TNF-α release from Caco-2 cells after 48 h exposure on AgBr-decorated NPs. In the control, free of NPs samples, cytokine level was below the detection limit of the test (mean ± SD, n = 2).

**a.**		**48 h-IC_50_ (μg/mL)**		SI *
		**CCD841CoN**	**Caco-2**	
CUR		nt	18.26 ± 0.01	-
CuMS		>800	>800	≤1
ApCuMS		>400	124.87 ± 0.018	≥3.2
CUR-CuMS		>800	>800	≤1
CUR-ApCuMS		>400	>400	≤1
AgMS		>200	34.48 ± 0.009	≥5.8
ApAgMS		103.76 ± 0.05	24.57 ± 0.021	4.2
CUR-AgMS		>200	46.93 ± 0.011	≥4.3
CUR-ApAgMS		74.53 ± 0.01	19.72 ± 0.032	3.8
**b.**		**TNFα/Caco-2**		
**Sample**	**AgMS**	**ApAgMS**	**CUR-AgMS**	**CUR-ApAgMS**
**μg/mL**	**pg/mL**			
6.25	LoD	LoD	LoD	19.3 ± 0.009
12.5	LoD	26.9 ± 0.009	LoD	38.2 ± 0.033
25	LoD	22.7 ± 0.01	25.7 ± 0.006	45.0 ± 0.002
50	54.6 ± 0.009	49.4 ± 0.009	31.9 ± 0.01	146.9 ± 0.019
100	nt	107.8 ± 0.052	64.3 ± 0.013	224.8 ± 0.05
200	nt	141.5 ± 0.023	50.7 ± 0.007	168.8 ± 0.028

* SI values are estimates based on available IC_50_ values, nt—not tested. LoD—limit of detection.

**Table 3:** RSA % of the tested nanomaterials at 100 µg/mL. Ascorbic acid (500 µg/mL) was used as reference, DPPH alone served as control, and CUR was tested at 5 µg/mL (n = 3).

Samples	OD517 (x̄ ± SD)	DPPH RSA (%)
Ascorbic acid	0.023 ± 0.003	89.15
Curcumin	0.052 ± 0.001	75.63
CUR-CuMS	0.113 ± 0.003	46.70
CUR-ApCuMS	0.108 ± 0.001	48.90
CUR-AgMS	0.112 ± 0.011	47.17
CUR-ApAgMS	0.124 ± 0.003	41.19
DPPH	0.212 ± 0.021	-

**Table 4:** Antimicrobial activity of tested materials presented as minimal inhibitory concentration (MIC) and minimal bactericidal/fungicidal concentration (MBC/MFC) (µg/mL).

Microorganism	Curcumin		AgMS		ApAgMS		CUR-AgMS		CUR-ApAgMS	
	MIC	MBC	MIC	MBC	MIC	MBC	MIC	MBC	MIC	MBC
Gram-positive bacteria										
*S. aureus* ATCC 25923	500	>1000	125	250	62.5	125	250	500	125	250
*S. aureus* ATCC BA	>1000	>1000	125	250	62.5	125	250	500	125	250
*S. epidermidis* ATCC 12228	500	500	62.5	250	62.5	125	125	500	62.5	250
*M. luteus* ATCC 10240	250	250	62.5	125	31.25	62.5	125	250	62.5	62.5
*B. cereus* ATCC 10876	250	>1000	250	>1000	62.5	1000	500	500	125	500
*E. faecalis* ATCC 29212	>1000	>1000	125	500	62.5	250	500	500	125	250
Gram-negative bacteria										
*S. typhimurium* ATCC 14028	>1000	>1000	250	500	250	250	500	500	125	250
*E. coli* ATCC 25922	>1000	>1000	125	250	31.25	250	125	250	125	250
*P. mirabilis* ATCC 12453	>1000	>1000	125	1000	125	250	500	500	250	500
*K. pneumoniae* ATCC 13883	>1000	>1000	250	500	125	250	125	250	125	250
*P. aeruginosa* ATCC 9027	>1000	>1000	62.5	500	62.5	500	250	500	62.5	250
Yeasts	MIC	MFC	MIC	MFC	MIC	MFC	MIC	MFC	MIC	MFC
*C. glabrata* ATCC 90030	125	>1000	62.5	125	31.25	125	125	250	62.5	250
*C. albicans* ATCC 102231	>1000	>1000	31.25	125	31.25	125	250	500	62.5	125
*C. parapsilosis* ATCC 22019	>1000	>1000	62.5	250	31.25	250	250	1000	125	125

## Data Availability

The datasets used during the study are available from the corresponding author upon request. Please contact the corresponding authors for the data requests.

## Supplementary Materials

The following supporting information can be downloaded at: https://www.mdpi.com/article/10.3390/nano16181163/s1, Figure S1: FT-IR spectrum of CUR, Figure S2: Kinetics of CUR degradation at 37 °C in buffer solutions with pH 5.4 and 7.3, Figure S3: Effect of the tested materials on the viability and proliferation of Caco-2 cells (left) and CCD841CoN (right) after 48 h exposure assessed by the XTT assay (mean ± SD, * significantly different from negative control at *p* < 0.05), Figure S4: Keto-enol tautomeric forms of curcumin, Figure S5: FAAS calibration curves of: (a) silver; (b) copper.
