# Supplementary material for: Synthesis of Modified Silver- or Copper-Loaded Silica Nanocarriers as a Tool for Curcumin Delivery
Source: Nanomaterials (Basel). 2026 Sep 15;16(18):1163. doi: 10.3390/nano16181163 (PMC13610866; doi:10.3390/nano16181163)
Supplement: Supplementary file 1 [file nanomaterials-16-01163-s001.zip › nanomaterials-4525262-supplementary.pdf]

*Supplementary materials*

# Synthesis of Modified Silver- or Copper-Loaded Silica Nanocarriers as a Tool for Curcumin Delivery

**Anna Sierosławska [1,*,†], Anna Borówka [1,*,†], Izabela Korona-Głowniak [2], Anna Rymuszka [1], Jakub Ciepielski [1] and Andrzej Górski [1]**

[1] Faculty of Medicine, The John Paul II Catholic University of Lublin, 20-708 Lublin, Poland
[2] Faculty of Pharmacy, Medical University of Lublin, 20-093 Lublin, Poland
* Correspondence: anna.sieroslawska@kul.pl (A.S.); anna.borowka@kul.pl (A.B.)
† These authors contributed equally to this work.

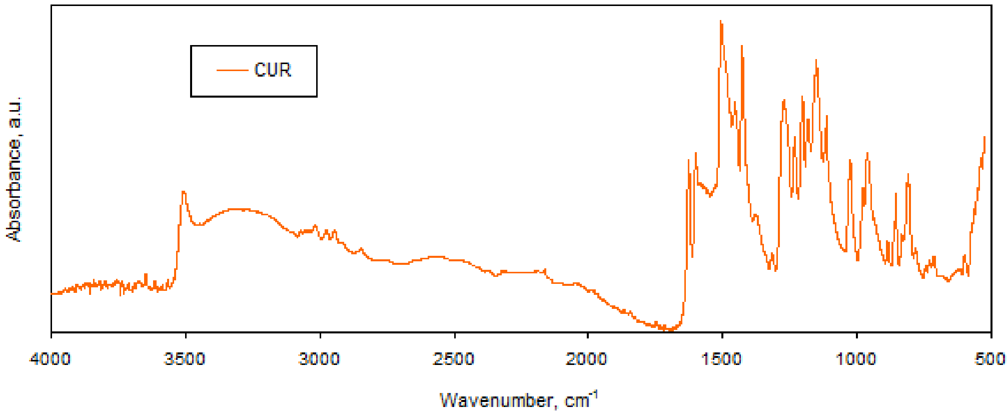


**Figure S1.** FT-IR spectrum of CUR.

In pure CUR spectrum (Fig. S1) strong bands, especially in the "finger print" region (500-1600 cm$^{-1}$) are clearly visible. The band at 1429 cm$^{-1}$ is attributed to >C=C< aromatic stretching vibration, while at 1509 to >C=C< aliphatic chain stretching vibration. An intense signal at 1280 cm$^{-1}$ is referred to the bending vibration of the C-O phenolic band and the band at 815 cm$^{-1}$ to the >C=CH- aromatic stretching vibration. The O-H groups give signals in the range 3200-3500 cm$^{-1}$, while at 3500 cm$^{-1}$ the stretching vibration O-H band is observed. A band characteristic of the carbonyl group is visible at 1630 cm$^{-1}$. However, its intensity is not hi**Figure S**gh, which indicates an enolic form of CUR with a hydrogen atom bonded to both carbonyls (Fig. S4).

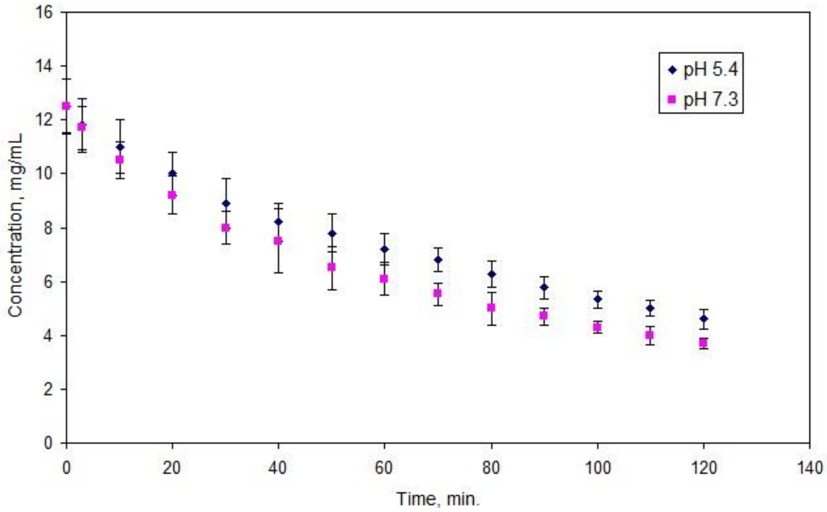


**Figure S2.** Kinetics of CUR degradation at 37°C in buffer solutions with pH 5.4 and 7.3.

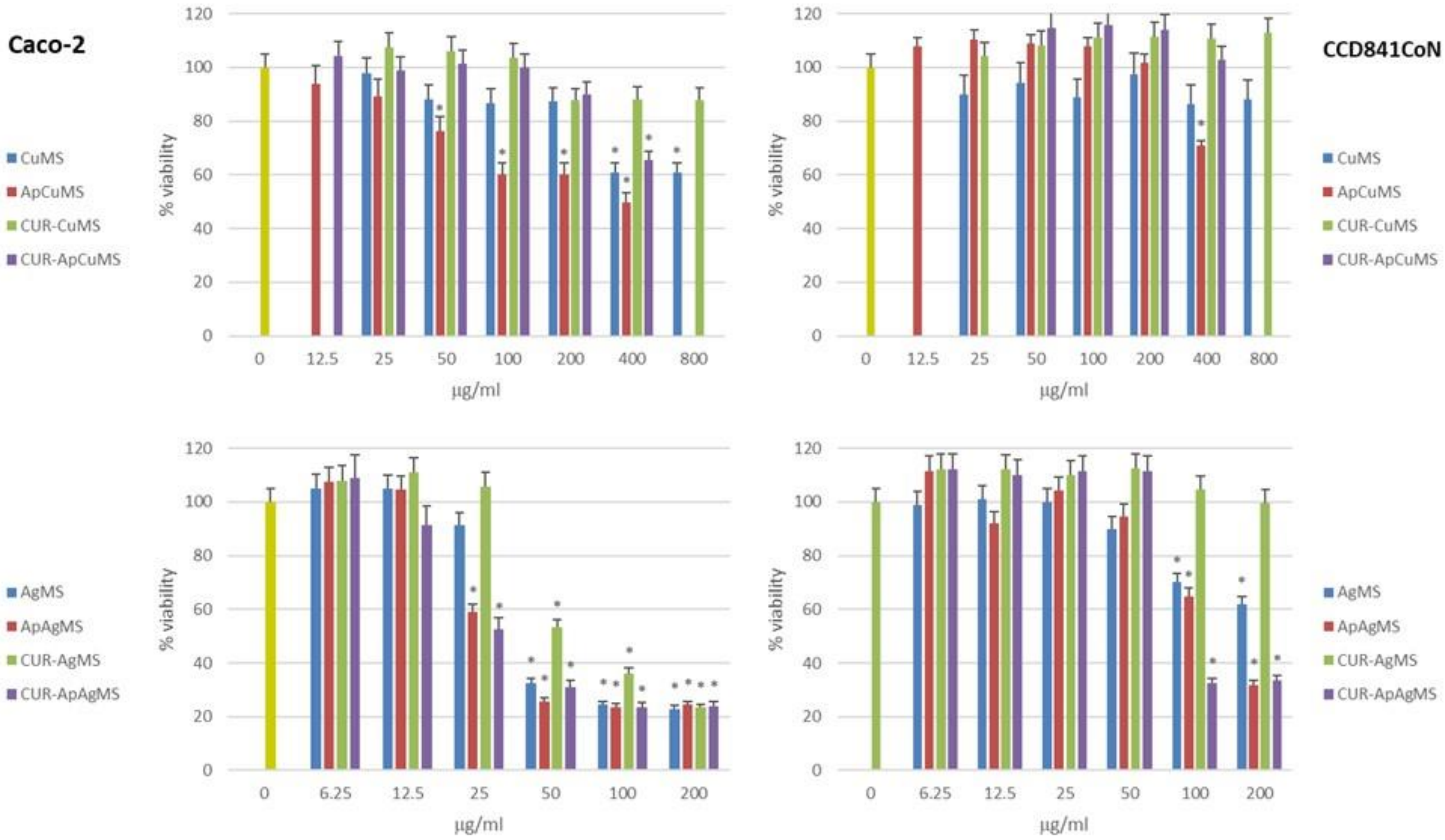


**Figure S3.** Effect of the tested materials on the viability and proliferation of Caco-2 cells (left) and CCD841CoN (right) after 48 h exposure assessed by the XTT assay (mean ± SD, * significantly different from negative control at $p < 0.05$).

Statistical analysis of the data shown on Fig. S3 was performed using Statistica 13.0 software (StatSoft Inc., Tulsa, OK, USA). One-way analysis of variance followed by Duncan's test was used. $P < 0.05$ was considered to indicate statistical significance.

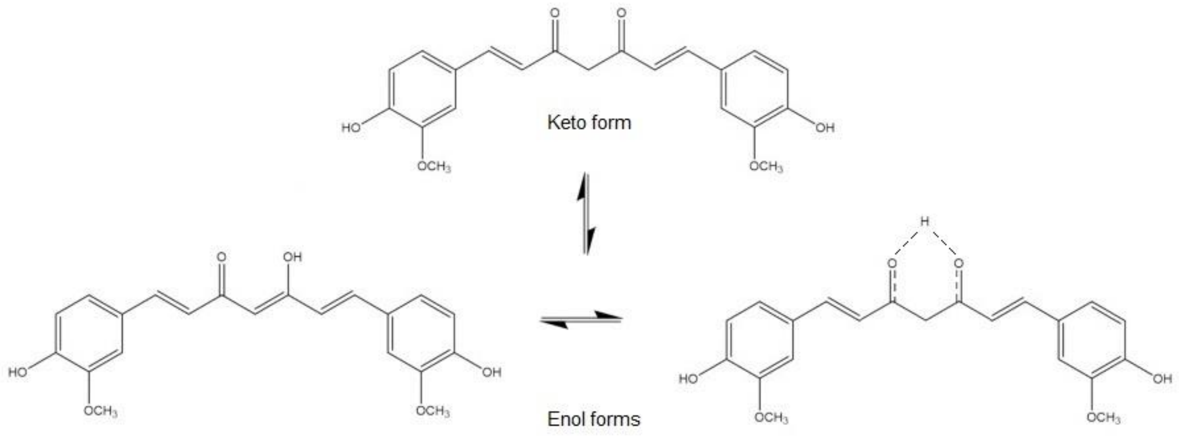


**Figure S4.** Keto-enol tautomeric forms of curcumin.

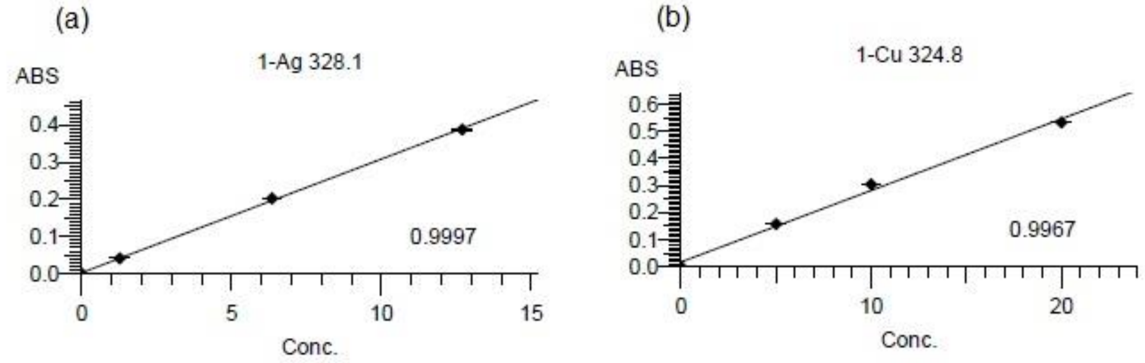


**Figure S5.** FAAS **c**alibration curves of: (a) silver; (b) copper.
